# Looking for Needles in the Plasmodial Haystack

**DOI:** 10.1128/mSphere.00146-19

**Published:** 2019-03-27

**Authors:** Lars Hviid

**Affiliations:** a Centre for Medical Parasitology, Department of Immunology and Microbiology, University of Copenhagen, Copenhagen, Denmark; b Department of Infectious Diseases, Copenhagen University Hospital (Rigshospitalet), Copenhagen, Denmark

**Keywords:** *Plasmodium falciparum*, antibodies, antigens, malaria

## Abstract

Plasmodium falciparum malaria remains a globally leading infectious disease problem. Despite decades of intense investigation, an efficacious and practical vaccine offering durable protection to people living in areas with transmission of malaria parasites remains an elusive goal. Our fragmentary understanding of the mechanisms of protective immunity to the disease is a major obstacle, and the almost complete focus on a very small subset of P. falciparum proteins as vaccine candidates has left most parasite antigens essentially unexplored as targets of acquired immunity. However, with the protein microarray technology, it is now possible to interrogate the entire parasite proteome for new vaccine candidates and for markers of parasite exposure. Recent *mSphere* papers describe the results of such research.

## Abstract (precis)

Plasmodium falciparum malaria remains a globally leading infectious disease problem. Despite decades of intense investigation, an efficacious and practical vaccine offering durable protection to people living in areas with transmission of malaria parasites remains an elusive goal.

## THE MALARIA PROBLEM

The most serious form of malaria, caused by the hemoprotozoan parasite Plasmodium falciparum, is a major cause of human suffering and death, not least in equatorial Africa. Although the number of cases has decreased steadily over the last decade, the pace is flagging, and the disease burden remains completely unacceptable (1). Continued progress toward eliminating and ultimately eradicating malaria is threatened by drug resistance, insecticide resistance, and other problems. An efficacious vaccine that is suitable for mass administration and that ideally induces durable protection after a few immunizations would be a major game changer. Indeed, such a vaccine has been the holy grail of malaria immunology research for decades. Regrettably, the reward of this eager pursuit has been meager, and the only malaria vaccine licensed so far affords only partial and short-term protection. One limitation of the vaccine development efforts has been the dogged focus on a few antigens (WHO, Malaria Vaccine Rainbow Tables [http://www.who.int/vaccine_research/links/Rainbow/en/index.html; accessed 26 February 2019]).

## THE PLASMODIAL HAYSTACK

Most of the current vaccine candidates were discovered many years ago and showed promise in initial work, but many are still backed by less than rock solid evidence that they are in fact key targets of protective immunity against P. falciparum malaria in humans.

In areas with stable parasite transmission, clinical immunity to P. falciparum malaria is acquired in a piecemeal manner over several years and after many infections and disease episodes. It is paralleled by acquisition of antibodies to many of the approximately 5,300 proteins encoded by the parasite (2). Suspected protective antigens may thus, in reality, be covariates of genuinely protective antigens, and separating the wheat from the chaff in this enormous plasmodial haystack has proved very difficult. The task is further complicated by the fact that many P. falciparum antigens are highly polymorphic and/or clonally variant. Finally, we cannot even be certain that there is indeed a particular immune response targeting a particular epitope in a particular antigen that mediates protection across the board.

## FINDING THE NEEDLE(S) IN THE HAYSTACK

Given the uncertainty that any of the relatively few antigens investigated thoroughly so far really holds the solution to the vaccine challenge—the needle in the above-mentioned haystack—it seems obvious to widen the portfolio of candidates. Until fairly recently, technology has seriously limited the practicality of comprehensive screening for novel clinically important candidates, but that may be about to change. The authors of a recent report in *mSphere* (3) thus combined the remarkable ability of controlled human malaria infection (CHMI) to induce protective immunity (4) with the possibility of identifying important antibody reactivities mediating it, by employing large protein arrays including thousands of antigens (5). The analysis by Obiero et al. (3) of an array covering more than 90% of the P. falciparum proteome revealed that in some individuals CHMI induced antibody responses to only a few antigens (low responders), broader and higher responses in others (medium responders), and high responses to many antigens in yet other CHMI recipients (high responders). Remarkably, essentially all low and high responders were clinically protected from challenge infection, suggesting that clinical protection can be achieved in different ways in different individuals, even when exposed to infection in exactly the same way. However, the authors focused on the moderate responder group, which contained most of the nonprotected individuals but also several who were protected. Analysis of the antibody responses among susceptible versus protected moderate responders allowed the authors to identify small subsets of antibody specificities associated with either status. Perhaps even more remarkable was the finding that the six protective antigens did not include any current vaccine candidate, whereas four of the six susceptibility markers belonged to members of the P. falciparum EMP1 (PfEMP1) family that have otherwise been implicated as important mediators of clinical protection after natural exposure (6). The study by Obiero et al. (3) thus illustrates the great potential of brute-force studies not only for identifying new vaccine candidates (“needles”) but also for inadvertently increasing the size of the plasmodial haystack to be sifted. More focused traditional follow-up studies are undoubtedly needed to confirm or refute the validity of the candidates identified by hypothesis-free approaches, such as the one used by Obiero et al. (3), and to delineate plausible protective mechanisms of promising antibody specificities. Hybrids between the new hypothesis-free all-encompassing approach and the traditional, hypothesis-driven single/few-antigen approaches might offer an interesting compromise. The KILchip merozoite antigen array (7) and the PfEMP1/RIFIN/STEVOR array (8) are examples of this.

## SEVERAL TYPES OF NEEDLES

The study of Obiero et al. (3) also illustrates another interesting point. Although particular antibody responses are not directly related to clinical protection, they can nevertheless sometimes be used as markers of short-term or long-term changes in exposure. Such responses can therefore be convenient tools to map malaria endemicity and to monitor the efficacy of interventions. Relevant antibody specificities for this task constitute a class of antigenic “needles” different from those discussed above and, arguably, one easier to find, not least by big antigen arrays, obviously so not only because of their comprehensiveness but also because fidelity in the reproduction of particular native epitopes is likely to be less of a concern than when looking for the targets of protective antibodies. In another recent *mSphere* report (9), Kobayashi et al. used a 500-exon array to analyze serum samples collected in various surveys at sites with different transmission intensities and patterns in southern Africa. While falling short of identifying a one-size-fits-all set of exposure markers, the study demonstrated important age- and transmission intensity-dependent qualitative and quantitative differences in antibody responses, similar to findings from previous studies (10–12). Furthermore, the study supports the idea that acquisition of protective immunity to malaria involves not only increases in antibody quantity and quality but also a gradual shift from predominantly transient responses in childhood to more sustained responses in adults, as proposed previously (13). Such a transition may be an important reflection of how immunological memory to malaria is induced and maintained.

## CONCLUDING REMARKS

Antigen arrays of steadily increasing comprehensiveness and quality are becoming powerful tools in the analysis of acquired immunity to P. falciparum malaria. The recent *mSphere* reports (3, 9) are good examples of this. Big antigen arrays clearly have the potential to help us identify sorely needed new and clinically decisive antigenic targets. However, if not used carefully—and probably best in conjunction with other, lower-throughput but more in-depth approaches—they may also generate a considerable number of false leads. We should certainly use these tools (we need all the help we can get) but must take care to use them wisely.
